# Editorial for the Special Issue on Microfluidics for Cells and Other Organisms

**DOI:** 10.3390/mi10080520

**Published:** 2019-08-05

**Authors:** Danny van Noort

**Affiliations:** Department of Physics, Chemistry and Biology (IFM), Linköping University, 581 83 Linköping, Sweden; drr.dvn@gmail.com

It is my great pleasure to present to you this first volume of 13 papers on the subject of Microfluidics for Cells and other Organisms. By adding “organisms” to the volume title, I was hoping for manuscripts beyond just cells. So, it was good to see that there were submissions of papers on zebrafish [1] and bacteria [2]. This volume highlights a diverse collection of research on single cell manipulation, diagnostics, cell migration, cell flow cytometry, to name a few. I am also happy to see that some papers included automated systems to operate the devices [3,4]. Automation is needed if we want to have a more intensive use of microfluidic based platforms and reproducibility.

Contributions to this volume came from all over the world, from Germany, France, Switzerland, USA, Hong Kong, China, Taiwan, Japan, Singapore and Chile.

This volume shows the importance of using microfluidics as a tool to understand cells and other organisms or even broader, biology better.

As Constantinou et al. [5] showed, hydrodynamic focusing inside a Y-shaped microfluidic device improve the classification of single cells in cytometry. With the aid of image analysis, cells can be identified in cell mixtures.

Analysis of nuclear acids is important for molecular diagnostics as well as automation of the process. Tong et al. [3] introduced a rotating disk device to extract the nuclear acid from cells using magnetic beads. 

From my own work on zebra fish embryos, I know how they easily evade the viewing field of the microscope when looking at them in a petri dish. Thus, I developed a chip to keep the embryos in place for real-time observation [6]. Zhu et al. [1] had the same idea but developed a different trapping method to keep the embryos in place.

Cells react to external forces, which is very well studied in the field of mechanobiology. By applying periodic hydrostatic pressure on cells, Horade et al. [7] showed that cells under periodic pressure displayed a faster increase in the size of the cells as compared to atmospheric pressure. Another example in the field of mechanobiology is given by Li et al. [8]. The biomechanical properties of cells can be used for early disease diagnostics. By using a microfluidic device like a Wheatstone Bridge, single cells could be trapped and exposed to precisely controlled pressures.

Performing diagnostics on prenatal fetuses is basically impossible, unless you can do it non-invasively by isolating some cells from the fetus. As it turns out, circulating fetal cells (CFCs) are present in the maternal blood. Ma et al. [4] designed a chip and an automated system to isolate this rare cell from the maternal blood.

Cheap and simple in-situ alternatives to standard flow cytometry is making its way to microfluidic devices. I see this as a positive development of more portable devices, which can be deployed remotely to, for example, perform diagnostics. Zhang et al. [2] used conductivity to measure the concentration of bacteria. 

Microfluidics can also be used to find binding proteins to specific cancer cells. It is a way to identify the cancer cell. Kaminaga et al. [9] did exactly that, by using micropillar arrays to filter non-target-binding-molecules from specific binding molecules. Liu et al. [10] looked at specific protein interaction on cells, however, at a single cell level. They are specifically looking for oral tumor cells from patients with their microfluidic based cell analyzer.

Single cell cultures do not have the same functionality as co-cultures. Chen et al. [11] fabricated a non-contact co-culture chip with fibroblasts and lung cancer cell lines to study their interaction, with the intention to explore the mechanism of cancer.

Another method to separate cells is to look at their motility, especially when looking at migrating cancer cells. Wang et al. [12] proposed to measure the motility of these cells to access the effect of anti-cancer drugs, by using a paper-based microfluidic device.

Single cell analysis is further highlighted in a review by Luo et al. [13]. It explores various methods for single cell manipulation, analysis as well as the various microfluidic devices available. 

Finally, this volume ends with an opinion piece by Grenci et al. [14] highlighting the role of microfluidics or more precise, the role of micro and nanotechnology in biological and biomedical applications. It describes the interdisciplinary processes to develop new biological technologies

Due to the success of this volume of papers, I am now looking forward to the contributions in Volume 2.
